# Evaluation of pH, Calcium Ion Release, and Dimensional Stability of an Experimental Silver Nanoparticle-Incorporated Calcium Silicate-Based Cement

**DOI:** 10.1155/2021/3919543

**Published:** 2021-12-03

**Authors:** Teena Sheethal Dsouza, Aditya Shetty, Neevan Dsouza

**Affiliations:** ^1^Department of Conservative Dentistry & Endodontics, AB Shetty Memorial Institute of Dental Sciences, NITTE (Deemed to be University), Mangalore, India; ^2^Department of Humanities, KS Hegde Medical Academy, NITTE (Deemed to be University), Mangalore, India

## Abstract

An experimental calcium silicate-based root-end filling material incorporated with silver nanoparticles intended for use in periapical surgeries was developed with the purpose to overcome the drawbacks of existing materials and to satisfy the ideal requirements of root-end filling materials. This study was designed to evaluate the physicochemical properties, pH, calcium ion release, and dimensional stability of the experimental cement, and compare the results with commercially available ProRoot MTA (Dentsply). An independent sample test was used to analyze the data. Mean initial pH (immediately after mixing) of the experimental cement was 10.42 ± 0.04 which was higher than that of MTA. However, there was a significant increase in pH of MTA at 1 day, 2 days, and 7 days. Presence of calcium chloride favored the release of calcium ions which was significantly increased in the experimental group at 24 hours. At the end of 30 days, MTA showed a significant expansion when compared to the experimental cement (*p* < 0.001). In conclusion, the experimental nanoparticle-incorporated calcium silicate-based cement showed clinically acceptable physicochemical properties.

## 1. Introduction

The success rate of a conventional root canal treatment is very high and nearly approaching 95%. On occasional reinfections, nonsurgical retreatment is performed. However, when there is failure of nonsurgical root canal treatment or when retreatment is not advised, treatment by a surgical approach involving root-end resection, retrograde cavity preparation, and filling is indicated to obtain a good apical seal and resolve persistent infections [1, 2].

The introduction of calcium silicate-based materials in endodontics has been an explicit discovery for use in blood and moisture-field periapical surgeries. With the advent of mineral trioxide aggregate in 1993 by Torabinejad, several calcium silicate- and bioceramic-based materials have been marketed. Bioceramic sealers such as EndoSequence BC Sealer, EndoSeal MTA, and MTA Fillapex have been appreciated for their favorable physicobiological properties [3, 4]. Calcium silicate-based cements have been developed as root-end filling materials mainly due to its hydraulic property which allows it to set even when in contact with tissue fluid and blood [5, 6]. Calcium silicate cements such as mineral trioxide aggregate (MTA) and other Portland-based cements have also shown promising results regarding biocompatibility and sealing ability [7]. Another notable feature and a booming interest in the potential clinical applications of hydraulic calcium silicate cements is their bioactivity that influences the surrounding environment [8, 9]. Shokouhinejad et al. proved that ProRoot MTA and EndoSequence root repair material were bioactive with increased effect over time [10]. A research study conducted by Gandolfi et al. in 2017 explored the ability of calcium silicate-based cements to enable biomineralization, with an unmediated bond between the surface of these materials and the mineralized bone matrix [11].

However, due to the inherent drawbacks in these cements including high cost, slower setting, and limited antibacterial activity, new additives have been added to the existing commercially available materials. Selected accelerants such as calcium chloride, calcium nitrate, and calcium formate have been added to improve the physical properties of MTA [2, 12, 13]. In an attempt to increase the bioactivity and early formation of fluorapatite, Gandolfi et al., in 2011, introduced doping of fluorides in calcium silicate cements [14]. Zamparini et al. added tantalum pentoxide and zirconium oxide into bioceramics to enhance apatite-forming ability with fulfillment of the desired chemical and physical standards [15]. Antimicrobial agents such as silver and gold nanoparticles and titanium dioxide nanoparticles have been used to inhibit bacterial growth [16, 17]. Samiei et al. incorporated 1 wt% silver nanoparticles to MTA in an attempt to improve the antibacterial activity with desirable results [18].

Nonetheless, there are limited studies in the literature that reveal any adverse changes in the chemical properties of calcium silicate-based cements when these accelerants and nanoparticles are added. One characteristic whose change would be of concern is the dimensional stability because it is likely related to its ability to seal the root-end [12]. Another attribute to deal with is the alkalinity of the calcium silicate-based cements. Alteration in pH might cause changes in the antibacterial activity and the release of calcium ions. Calcium ions are desirable for hard tissue formation [12]. Neither scenario would be clinically acceptable. The purpose of this study was to evaluate the influence of adding calcium chloride and silver nanoparticles in terms of pH, dimensional stability, and calcium ion release on a low-temperature production-based experimental calcium silicate-based cement.

## 2. Materials and Methods

A series of experiments were initially performed to determine the most appropriate concentrations of the ingredients of Portland cement that best improved cohesiveness and workability. The components included 60% calcium oxide, 20% silicon dioxide, and 9% aluminium oxide. Other additives included bismuth oxide as a radiopacifier and 1 wt% silver nanoparticles (silver nanopowder, Type 2, APS: 20 nm, SRL Chemical) for antimicrobial action. 10% calcium chloride solution was used as the liquid component to accelerate the setting time. This was referred to as the experimental cement.

The powder components were mixed using the solid-state reaction technique using ethanol as a solvent. The resultant powder was further subjected to sintering using a hot air oven at 100°C for 24 hours. The final powder was passed through a 250-micron mesh to attain a desired homogenous powdered mixture. The experimental cement was subjected to further analysis. A pilot study was conducted to test the antibacterial activity using agar diffusion test on *E. faecalis* which exhibited a zone of inhibition of 3-4 mm. The accelerated setting of the cement was evaluated by preliminary tests using a Gillmore needle apparatus which confirmed a shorter setting time of 6-7 min as reported by Dsouza et al. [19]. On comparison, ProRoot MTA showed a delayed setting time of 170 min. Elemental analysis was performed, and powder microstructure was evaluated to confirm the presence of oxides and silver nanoparticles in the final powder using energy-dispersive analysis by X-rays (EDAX) in the scanning electron microscope (Zeiss Gemini).

### 2.1. Groups

  Group 1: experimental cement
  Group 2: mineral trioxide aggregate (ProRoot MTA, Dentsply Sirona)

### 2.2. pH Measurement

Polyethylene tubes (3 mm in diameter and 1 mm length) were used. The powder (0.2 g) and liquid (0.14 mL) were mixed to get a desired putty consistency. After filling the tubes with the material, they were immersed in containers containing 10 mL of deionized water, and initial pH was immediately noted. Subsequent readings were made after 1 day, 2 days, and 7 days. A digital pH meter (Merck Life Science Pvt Ltd.) was used for pH measurement. The apparatus was previously calibrated with pH 7.0 ± 0.05 buffer capsules. Between each measurement, the electrode was washed with ultrapure water and blot-dried. Initial pH of deionized water was measured before the immersion of the materials. A similar procedure was followed to determine pH of ProRoot MTA (Dentsply).

### 2.3. Calcium Ion Release Analysis

Polyethylene tubes with 10 mm length and 1 mm internal diameter were used. The filled tubes with the experimental cement were immersed in 10 mL of deionized water. The calcium ion release profile from the cement samples was recorded at day 1 and day 7 using an atomic absorption spectrophotometer (GBC 932 Plus), equipped with a hollow calcium cathode lamp, according to the following operative conditions: lamp current 3 mA; fuel nitrous oxide; support oxygen; stoichiometry reducing; wavelength 422.7 nm; slit 02 nm. The results were estimated by using the equation of standard curve line, which was determined by the measurement of solutions with known concentrations. The calcium ion release of ProRoot MTA was also determined.

### 2.4. Test of Dimensional Stability

This test was based on ISO 6876:7.6 with split molds (height 6 mm and width 3 mm). After mixing, the samples were transferred to the molds until slightly overfilled. Filled molds were flattened on each end, wrapped in a water-moistened gauze, and placed in a 37°C incubator with humidity maintained at 95%. After 48 hours, the samples were drawn across fresh 600-grit sandpaper to flatten their ends. The samples were removed from the molds, their lengths were measured using a digital caliper with a resolution of 0.01 mm, and they were stored in distilled water at 37°C. After 30 days, their lengths were measured again. The change in length during a period of 30 days divided by the original length was our measure of dimensional stability. The test was repeated 3 times. In accordance with the ISO and ANSI/ADA, the results must not exceed 1.0% of contraction or 0.1% of expansion.

## 3. Results

### 3.1. EDAX SEM Analysis

**Figure 1:**
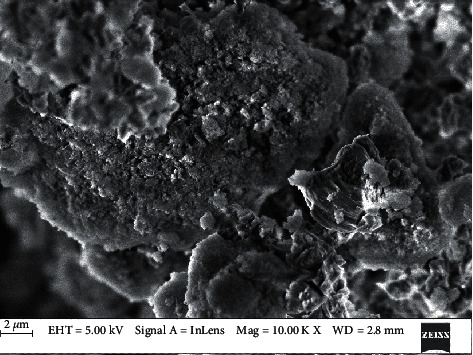
Powder microstructure under the EDAX SEM.

Elemental analysis confirmed the presence of oxides of the powders with pronounced peaks for calcium, silicon, and oxygen. Powder microstructure of the unhydrated cement showed coarse and irregular particles under energy-dispersive analysis by X-rays (EDAX) in the scanning electron microscope (Figure 1).

### 3.2. Evaluation of pH and Calcium Ion Release

Throughout the experimental period, the results of pH measurements of both cements were alkaline. Independent sample test showed a statistically significant difference which was observed in immediate pH as well as pH at 1 day, 2 days, and 7 days between the two groups. Mean pH of the experimental group in the immediate period was 10.42 ± 0.04 which was higher than MTA (group 2). However, there was a significant increase in pH of group 2 (MTA) at 1 day, 2 days, and 7 days compared to group 1 (*p* < 0.05) (Table 1).

Presence of calcium chloride favored the release of calcium ions which was significantly increased in group 1 at 24 hours (*p* < 0.001). However, no significant difference was noted at 7 days between group 1 and group 2 (*p*=0.698) (Table 2).

### 3.3. Evaluation of Dimensional Stability

At the end of 30 days, group 2 showed a significant expansion when compared to group 1 (*p* < 0.001) (Table 3).

## 4. Discussion

Hydraulic calcium silicate cements have been a boon to the clinicians as alternative biomaterials for dentine replacement, in vital pulp therapy, apexogenesis and apexification, root perforation repair and resorptive defects, root canal, and root-end restorations [8, 15].

This study evaluated the physicochemical properties of a low-temperature fabricated calcium silicate-based cement. There were pronounced peaks of calcium and silicon in the EDAX analysis which were similar to the study results conducted by Gandolfi et al. They confirmed that calcium silicate materials, i.e., ProRoot MTA, MTA Plus, and Biodentine, also showed an increase in calcium and phosphorus that was necessary for biomineralization [11]. Concordance to our study, Shokouhinejad et al. analyzed the elemental composition of calcium silicate-based EndoSequence root repair material, BioAggregate, and ProRoot MTA with display of high peaks for calcium [10].

On physicochemical evaluation, the addition of calcium chloride and silver nanoparticles to the experimental calcium silicate-based cement showed favorable results. In terms of pH, alkaline pH was maintained throughout the study period. The rich concentration of calcium oxide is converted into calcium hydroxide in an aqueous solution. Calcium hydroxide dissociates into calcium and hydroxyl ions, thus increasing pH of the solution and thus contributing to antibacterial action [20]. Alkaline pH is required as it has proven to offer osteogenic potential, biocompatibility, and antibacterial ability [4]. Initial pH after mixing was similar to that of MTA. However, variations in pH were seen at different time intervals between the experimental cement and MTA. At the end of 7 days, pH was reduced in the experimental group, but alkalinity was still maintained. The results of MTA were in accordance to a long-term study conducted by Fridland and Rosado who reported that usually reported pH of 12-13 may slightly decrease over time [21]. Thus, an additional ingredient of imparting an antibacterial action would be necessary to maintain a bacteria-free environment. This purpose was fulfilled by adding silver nanoparticles, the results of which were validated during the pilot study. Samiei et al. assessed the antimicrobial activity of MTA with silver nanoparticles with favorable results [18]. Silver nanoparticles (Ag NPs) are one of the most widely used nanoparticles, most notably serving as an antimicrobial agent for medical applications [22, 23]. Small-sized Ag NPs can inhibit the growth of nitrifying bacteria more than that by silver ions at the same total silver concentrations [24, 25]. The size of the particle was also related to antimicrobial activity; the smaller particles give more bactericidal effects compared to larger particles [26–28]. Gomes-Filho et al. reported that Ag NP dispersion was biocompatible, mainly at low concentrations [29].

Additional calcium incorporated into the experimental cement via the calcium chloride liquid boosted the calcium ion release. These findings further led to the assumption with the study results by Takita et al. that the continuous release of calcium ions is required for the proliferation of human dental pulp cells in calcium silicate-based cements [30]. A known fact is the formation of an interfacial calcium phosphate layer that forms a chemical bond between calcium silicate-based materials and walls of dentin [3]. Also, these are placed directly in contact with periradicular tissues; therefore, an adequate response of bioactivity is much desired [14]. The amount of calcium ions released from the experimental cement was enhanced in 24 hours and almost similar to that of MTA after 7 days. Calcium chloride alone has low pH of 4.4, and its addition to experimental cement did alter pH. However, it did not seem to alter significantly the calcium ion release of the cement. This is in accordance with the findings reported by Antunes Bortoluzzi et al. which revealed that the addition of calcium chloride improved the calcium release properties of commercially available MTA cements [31]. Contradictory to the pH results of this study, Vazquez-Garcia et al. evaluated the radiopacity, setting time, pH, compressive strength, and solubility of Portland cement incorporated with silver nanoparticles and concluded that the addition of Ag NPs to PC/ZrO_2_ maintained pH, lowered the solubility, and increased the setting time and compressive strength [32].

The dimensional stability of the experimental cement was favorable. In accordance with the ISO and ANSI/ADA, the results must not exceed 1.0% of contraction or 0.1% of expansion. The experimental cement did not exceed 1% of contraction than the original dimension; however, MTA showed slight expansion which was also acceptable. In an in vitro dye leakage study conducted by Bortolluzzi et al., calcium chloride actually improved the sealing ability of MTA placed as a root-end filling material when compared with the control [33]. The dimensional stability of samples set with calcium chloride in this study supports the study results of Bortoluzzi et al. in that they exhibited no contraction. In another bacterial leakage study conducted by Dsouza et al. in 2016, incorporation of calcium chloride to MTA as a substitute for distilled water showed better sealing ability than when mixed with distilled water, chlorhexidine, and doxycycline [2]. The favorable dimensional change in this experiment may be related to the lower porosity of the cement and due to the occupancy of the silver nanoparticles into the empty spaces of the cement matrix [32].

## 5. Conclusion

In conclusion, the experimental calcium silicate-based cement with the incorporation of silver nanoparticles and calcium chloride had favorable physicochemical properties that can be useful to maintain a bacteria-free environment and for improved healing, which are necessary in root-end surgeries. Further investigations are indicated to assess the biological properties and the long-term sealing ability.

## Figures and Tables

**Table 1:** Comparison of pH analysis between the groups.

	Mean ± SD		*p* value	95% confidence interval of the difference	
	Group 1	Group 2		Lower	Upper
Immediate	10.42 ± 0.04	10.27 ± 0.06	0.001	0.12	0.19
24 hours	10.29 ± 0.06	11.88 ± 0.12	<0.001	−1.66	−1.52
48 hours	9.78 ± 0.11	11.47 ± 0.45	<0.001	−1.93	−1.44
7 days	8.90 ± 0.18	9.82 ± 0.05	<0.001	−1.01	−0.81

**Table 2:** Comparison of calcium ion release between the groups.

	Mean ± SD		*p* value	95% confidence interval of the difference	
	Group 1	Group 2		Lower	Upper
24 hours	15.92 ± 2.37	11.02 ± 0.92	<0.001	3.56	6.23
7 days	17.71 ± 5.91	18.30 ± 0.35	0.698	−3.73	2.56

**Table 3:** The dimensional stability (%) of the tested sealers at 30 days (mean ± standard deviation).

	Mean ± SD	
	Group 1	Group 2
Baseline (0^th^ day)	6.00 ± 0.00	6.00 ± 0.00
30 days	5.99 ± 0.06	6.00 ± 0.04

## Data Availability

The data used to support the findings of this study are included within the article.
